# The Mechanical Hypothesis as to the Origin of Cancer

**Published:** 1900-01-27

**Authors:** 


### THE MECHANICAL HYPOTHESIS AS TO THE ORIGIN OF CANCER.

At the last meeting of the Pathological Society Mr. Samuel G. Shattock showed a specimen of carcinoma of the uterus of the rabbit which was obtained from an animal upon which no kind of experiment had been performed. Histologically the growths were of the columnar-celled kind, in places papilliferous. The interest of the specimen lay in the bearing it had upon the experiment put on record by Dr. Lambert Lack in the *Journal of Pathology* for August, 1899. (See also The Hospital, July 22, 1899.) This experiment had been carried out by laying open the ovaries of a rabbit, scraping the surfaces, and allowing the milky juice containing free epithelial cells to diffuse into the peritoneal cavity, the laparotomy wound being, of course, afterwards closed. The animal remained well for nearly a year; it then became thin and weak, and suffered from dyspnœa; two months later it was killed, when the abdominal cavity was found studded with carcinomatous foci, other foci being found also in the thorax. The uterus was greatly thickened, its mucosa papilliform, and at one place in its wall was a tumour as large as a cherry. This, said Mr. Shattock, is the experiment, and the hypothesis is that the displacement of normal epithelium into a lymph space or lymph cavity is sufficient to bring about the growth of a carcinoma. A most ominous feature in the post-mortem finding, however, was the condition of the uterus, for the state of the uterine mucosa was precisely the same as that in the specimen exhibited by Mr. Shattock, in which it was easy to trace the source of the carcinoma of the uterine wall from the morbid mucous membrane. In coming to his conclusion as to the causal relationship between his experiment and the disease found after death, it would appear that Dr. Lack was influenced largely by the statement of Professor J. McFadyean that he had never seen or heard of carcinoma in the rabbit; but the specimen exhibited showed that carcinoma occurs in the rabbit, and the similarity of the uterine changes and of the structure of the carcinomata in the two cases seems to render it imperative that before deductions are drawn Dr. Lack's experiment should be repeated; for unless the striking result of this experiment can be obtained again it must be passed by as a coincidence—remarkable enough, indeed, but not entirely without parallel. This is a question in regard to which everything would seem to depend upon the number rather than upon the aptness of the observations, for if carcinoma be a mere mechanical infection of the lymph spaces by normal epithelium it should be possible to produce it as often as required. Clearly if the rabbit in Dr. Lack's experiment were at the time suffering from primary uterine carcinoma, no essential relationship would exist between the scraping of the ovary and the appearance of the intraperitoneal growths, and the experiment would not support the hypothesis. Moreover, there are other possible sources of fallacy. In any case the experiment should be repeated, and this Mr. Shattock said he had already done with several variants. Time only is wanted for the results to show themselves.

